# Threshold Responses of Forest Birds to Landscape Changes around Exurban Development

**DOI:** 10.1371/journal.pone.0067593

**Published:** 2013-06-24

**Authors:** Marcela Suarez-Rubio, Scott Wilson, Peter Leimgruber, Todd Lookingbill

**Affiliations:** 1 University of Maryland Center for Environmental Science, Appalachian Laboratory, Frostburg, Maryland, United States of America; 2 Canadian Wildlife Service, Environment Canada, Saskatoon, Canada; 3 Smithsonian Conservation Biology Institute, Front Royal, Virginia, United States of America; 4 University of Richmond, Department of Geography and the Environment, Richmond, Virginia, United States of America; Southern Illinois University, United States of America

## Abstract

Low-density residential development (i.e., exurban development) is often embedded within a matrix of protected areas and natural amenities, raising concern about its ecological consequences. Forest-dependent species are particularly susceptible to human settlement even at low housing densities typical of exurban areas. However, few studies have examined the response of forest birds to this increasingly common form of land conversion. The aim of this study was to assess whether, how, and at what scale forest birds respond to changes in habitat due to exurban growth. We evaluated changes in habitat composition (amount) and configuration (arrangement) for forest and forest-edge species around North America Breeding Bird Survey (BBS) stops between 1986 and 2009. We used Threshold Indicator Taxa Analysis to detect change points in species occurrence at two spatial extents (400-m and 1-km radius buffer). Our results show that exurban development reduced forest cover and increased habitat fragmentation around BBS stops. Forest birds responded nonlinearly to most measures of habitat loss and fragmentation at both the local and landscape extents. However, the strength and even direction of the response changed with the extent for several of the metrics. The majority of forest birds’ responses could be predicted by their habitat preferences indicating that management practices in exurban areas might target the maintenance of forested habitats, for example through easements or more focused management for birds within existing or new protected areas.

## Introduction

The expansion of human settlement along the urban-rural fringe has received considerable global attention in recent decades [1]–[5]. In the United States, conversion of privately owned rural lands into low-density residential development (i.e., exurban development) has increased five- to sevenfold between 1950 and 2000 [6]. In the Mid-Atlantic region of the United States, the dispersed, isolated housing units typical of exurban areas are embedded within a forest matrix, often close to protected areas [7] and natural amenities [8], [9]. Understanding the impacts of exurban development on wildlife and biodiversity is crucial to better understand long-term effects of exurban development and to develop successful land use and conservation planning [10], [11].

Humans generally remove natural habitats by building settlements, which can serve to fragment the landscape [12]–[14]. Both habitat loss and fragmentation modify the spatial pattern of remnant habitats, creating smaller and isolated fragments, thus compromising habitat quality and quantity. Wildlife responds in a variety of ways depending on species traits and life histories [15], [16]. Some species thrive in these environments whereas others, such as forest birds, decline rapidly (e.g., [17], [18]). Possible reasons for long-term reductions of forest-bird species in these environments include predation [19], brood parasitism [20], and competition with human-adapted species [21]. Forest birds have been shown to be particularly susceptible to human settlement even at housing densities as low as 0.095 house/ha [22]–[27].

Understanding how exurban development alters forest birds’ habitat over time is a conservation priority given the unprecedented rates of exurban development in eastern temperate forests of the Mid-Atlantic [6], [28]. Forest bird abundance is generally positively related to proportion of forest cover (e.g., [29], [30]), but the spatial distribution of suitable habitat also affects forest birds’ occurrence and fecundity [31], [32]. Declines of forest birds have been well documented in eastern North America, and these declines have been associated with habitat loss and fragmentation due to roads, power lines, and residential development [11], [33], [34]. However, few studies have examined the response of species through time as residential development progresses [18].

Species may respond nonlinearly to habitat loss and fragmentation (reviewed by [35]). Nonlinear responses of species to habitat loss and fragmentation may complicate our ability to determine the response of biodiversity to exurban development. Theoretical models predict the existence of a change point or threshold in which an abrupt reduction in occupancy occurs despite the presence of sufficient suitable habitat [36]–[39]. Some studies show empirical evidence for threshold existence in birds [40]–[43], although others have not found any evidence to support threshold responses [44]. It is uncertain whether threshold declines in forest birds apply to exurban development. If these relationships are appropriately characterized by threshold models, determining the range at which exurban development induces population crashes may provide guidance for landscape planning, management, and conservation.

The aim of this study was to assess whether and how forest birds respond to changes in habitat due to exurban growth. We evaluated habitat composition (amount) and configuration (arrangement) for selected bird species (i.e., forest and forest-edge species) around North America Breeding Bird Survey stops between 1986 and 2009. The approach accounted for year-to-year variability in species abundances and investigated species responses to both habitat loss and fragmentation as exurban development increased over time. In addition, we assessed whether selected bird species showed thresholds in both occurrence frequency and relative abundance. We used Threshold Indicator Taxa Analysis [45] to detect change points in species occurrence. We evaluated two spatial extents (400-m and 1-km radius buffer) to determine if species responded differently to changes at the local and landscape scales. We expected that forest species would exhibit a strong negative response to exurban development at both extents, whereas forest-edge species would respond positively to high levels of exurban land cover.

## Methods

### Study area

**Figure 1:**
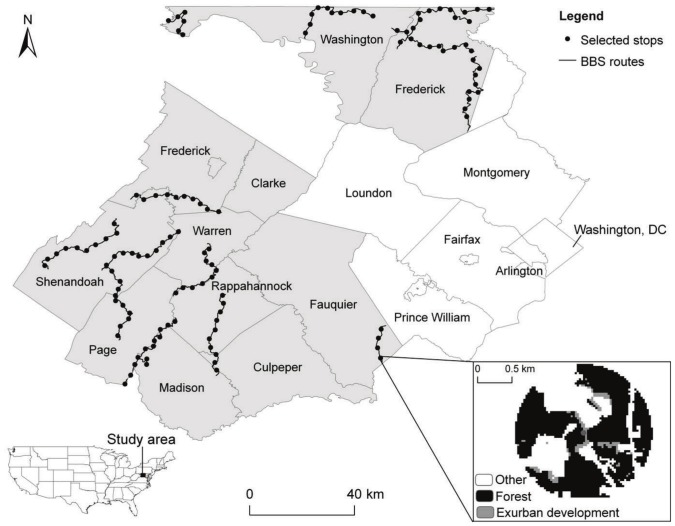
Study region (shaded area). It includes nine counties in north-central Virginia and two in western Maryland. Circles represent 125 North American Breeding Bird Survey (BBS) stops that were uniformly selected from routes. Zoom-in window shows example of a landscape within a 1-km radius of a selected survey stop.

The study area encompassed nine counties in north-central Virginia (Clarke, Culpeper, Fauquier, Frederick, Madison, Page, Rappahannock, Shenandoah, and Warren) and two in western Maryland (Washington and most of Frederick; Figure 1). The region has experienced a remarkable population growth. For example, counties included in the study area had growth rates ranging from 4% (Page County) to 40% (Culpeper County) between 2000 and 2009 [46]. Concomitant with this population growth, the region has also experienced an increase in exurban area from 2.3% in 1986 to 7.3% in 2009 [28]. One reason for the increased exurban development is the easy access and well-maintained transportation infrastructure to the metropolitan Washington, DC area which provides employment opportunities [47].

### Breeding bird survey

We used the North America Breeding Bird Survey (BBS; [48], [49]) to gather relative abundance data. The BBS is a large-scale annual roadside survey to monitor the status and trend of breeding bird populations in the United States and southern Canada since 1966. The survey is performed along secondary roads by experienced volunteer observers in late May to early July, the peak of the breeding season. Routes are 39.4 km long and consist of 50 survey stops located at 0.8 km intervals. During the survey, observers record all birds heard or seen within 0.4 km in a 3-min period. We focused our analysis on survey stops instead of the entire route because our interest was on local characteristics of breeding habitats and routes might vary in local environmental conditions [50], [51]. We chose all routes located in the study area and from them we uniformly selected at most 10 survey stops per route (every fifth stop along the route). We only considered survey stops that had detailed direction descriptions and fell within the study region (125 survey points in total; Figure 1). This information was important for geocoding and characterizing site-specific features of selected survey stops. A maximum of 10 stops per route was chosen to reduce overlap between circular areas around survey stops and decrease the likelihood of spatial autocorrelation.

We focused on 11 forest-nesting passerine species whose habitat preferences included forest –Ovenbird (*Seiurus aurocapilla*), Red-eyed Vireo (*Vireo olivaceus*), American Redstart (*Setophaga ruticilla*), Wood Thrush (*Hylocichla mustelina*), Scarlet Tanager (*Piranga olivacea*), Eastern Wood-Pewee (*Contopus virens*), Eastern Phoebe (*Sayornis phoebe*); and forest-edge –Eastern Towhee (*Pipilo erythrophthalmus*), Gray Catbird (*Dumetella carolinensis*), Northern Cardinal (*Cardinalis cardinalis*), and Indigo Bunting (*Passerina cyanea*) [52]. We defined forest species as birds that utilized a wide variety of deciduous and mixed deciduous-coniferous forest types and that may favor interior forested habitats. Forest-edge species are those species that are strongly associated with forest edges and open habitats [53]. These species were selected to represent contrasting habitat preferences (forest vs. edge) and because they were detected on at least 5% of surveys during the 1986–2009 interval. In addition, many of these species are reported to have experienced population declines or reduced fecundity in their distribution range due to habitat loss or fragmentation [32], [54]–[56]. Our study was designed to determine if the specific land conversion process of exurban development corresponded with abundance changes for these species.

### Landscape structure around Breeding Bird Survey stops

We established circular areas of 400-m and 1-km radius around selected BBS stops. These areas were chosen to characterize both breeding bird territories [57], [58], which were assumed to be in the immediate surroundings of survey stops, and areas feasibly visited during bird daily movements [59], [60]. To quantify landscape structure around selected survey stops over time at these two extents, we used Landsat 5 TM imagery for 1986, 1993, 2000, and 2009. We performed standard pre-processing procedures (atmospheric and topographic correction) prior to image classification.

We used aerial photos to generate a training dataset to supervise a classification of areas of exurban development. Exurban development was defined as areas with housing densities between 1 unit per 0.4 ha and 1 unit per 16.3 ha (e.g., 6 - 250 houses per km^2^) [6]. We used both spectral and spatial characteristics to define and identify exurban areas [28]. Spectral characteristics were derived from spectral mixture analysis [61] of corrected Landsat images to estimate the fractional cover of vegetation, substrate, non-photosynthetic vegetation, and shade within each image. We built decision trees based on spectral mixture analysis outputs to classify exurban development between 1986 and 2009. We used morphological spatial pattern analysis to further analyze terminal nodes from the decision trees that could not discriminate between exurban and urban areas based on spectral characteristics alone [62], [63]. Scattered, isolated pixels were regarded as spatial characteristics typical of exurban development. This procedure allowed us to distinguish exurban areas from forest and urban areas and create a land-cover map that was used to characterize areas around survey stops.

We used FRAGSTATS 3.3 [64] and GUIDOS 1.3 [62], [63] to estimate both landscape composition and configuration within the two circular areas around selected survey stops for 1986, 1993, 2000, and 2009. Landscape composition variables described the amount of habitat and included proportion of area occupied by forest and exurban development. Landscape configuration variables described the arrangement of forest habitat and included area-weighted average patch size, number of forest patches greater than 0.45 ha, and proximity index [65]. Proximity index is a measure of isolation that considers both patch size and proximity of a focal patch to all forest patches around. We only considered forest patches ≥ 100 ha within 2500 m of the focal patch. A 2500 m range was selected to reflect dispersal patterns of most songbirds (dispersal median distance range: 0.3 – 7.3 km; [66]). The proximity index increases as the neighborhood is increasingly occupied by forest patches and as those patches become closer and more contiguous or less isolated. GUIDOS was used because it identifies and graphically depicts the different types of landscape elements created by the fragmentation process [63]. The software package analyzes map geometry by applying mathematical morphological operators to allocate each pixel to one of a mutually exclusive set of classes. We quantified changes in the proportion of forest interior (core class), forest fragments (islet class), and forest edge (edge and perforation classes).

Although some of these variables are not independent, many have been shown to affect abundance of birds [32], [67]–[70] and represent different aspects of potential habitat alteration.

### Analysis

BBS data have unknown precision due to observer differences [71], first-year observers’ skills [72], [73], environmental conditions [74], and habitat features [50]. We used a hierarchical Bayesian model to adjust BBS counts and account for these limitations. We modeled count data as hierarchical over-dispersed Poisson variables and fit models using Markov Chain Monte Carlo (MCMC) methods in WinBUGS 1.4.3 [75]. Hierarchical Bayesian models are frequently applied to BBS data [76]–[78] and are better able to account for variability in complex time series than other methods [79]. We specified *C_it_* as the count for each species on stop *i* and time *t* where *i*  = 1,..., N; *t*  =  1,…, T; and N and T were the number of stops and the number of years species were observed, respectively. Conditioned on the model, counts (*C_it_*) were independent across years and stops, and these conditional distributions for *C_it_* were assumed to be Poisson with mean μ*_it_:*

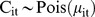
(1)


The full model was then:
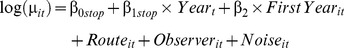
(2)where each stop was assumed to have a separate intercept (β_0_) and time trend (β_1_). The model also included several sources of variability including unknown route-level effects (*Route_it_*), observer effects (*Observer_it_*), and an additional noise component (*Noise_it_*) to help account for over-dispersion in the data. BBS observers tend to over or under-record certain species in their first year relative to subsequent years [77], [80] and to incorporate this effect we treated an individual’s first year (*FirstYear_it_*) as a binary indicator variable (β_2_). The precision parameters (τ*^2^*) for β_0-2_, observer, route, and noise effects were assigned vague inverse gamma prior distributions [81] with parameters (0.001, 0.001).

We used two Markov chains for each model and examined model convergence and performance through Gelman-Rubin diagnostics and individual parameter histories [82], [83]. Time to convergence varied among species depending on the amount of data for that species (30,000 – 200,000 iterations required). Once convergence was reached, we obtained derived estimates of the count at each stop and in each year, and these adjusted counts were then used for the threshold analysis. In addition, we estimated for each selected species the linear trend coefficient (i.e., the slope of abundance over time on a log scale) and percent annual change (the expected count in the last year divided by the expected count in the first year raised to 1/number of years). For trend coefficients (slope and percent annual change), we interpreted significance based on values with 95% credible intervals not overlapping zero.

We examined the relationship between landscape variables and selected species adjusted counts by fitting a non-parametric locally weighted polynomial regression (loess; [84]). When the loess regression highlighted nonlinearity in the relationship, then a change-point analysis to test for nonlinear threshold response was used.

We estimated potential species thresholds to landscape variables in space and time using Threshold Indicator Taxa ANalysis (TITAN; [45]). TITAN identifies abrupt changes in both occurrence frequency and relative abundance of individual taxa along an environmental gradient. It is able to distinguish responses of individual taxa with low occurrence frequencies or highly variable abundances and does not assume linear response along all or part of an environmental gradient. TITAN uses normalized indicator species taxa scores (z) to establish a change-point location that separates the data into two groups and maximizes association of each taxon with one side of the partition. Z scores measure the association of taxon abundance weighted by their occurrence and is normalized to facilitate cross-taxa comparison. Thus, TITAN distinguishes negative (z-) and positive (z+) indicator response taxa.

To measure quality of the indicator response and assess uncertainty around change-point locations, TITAN bootstraps the original dataset and recalculates change points with each simulation. Uncertainty is expressed as quantiles of the change-point distribution. Narrow intervals between upper and lower change-point quantiles (i.e., 5 and 95%) indicate nonlinear response in taxon abundance whereas broad quantile intervals are characteristic of taxa with linear or more gradual response. Diagnostic indices of the quality of the indicator response are purity and reliability. Purity is the proportion of bootstrap replicates that agree with the direction of the change-point for the observed response. Pure indicators (purity ≥ 0.95) are those that consistently assign the same response direction during the resampling procedure. Reliability is the proportion of change-point individual value scores (IndVal) among the bootstrap replicates that consistently have p-values below defined probability levels (0.05). Reliable indicators (reliability ≥ 0.95) are those with consistently large IndVal. Because purity and reliability indices did not differ for most metrics, we only reported the reliability index. We ran TITAN for the 11 selected bird species and each of the landscape variables in R 2.11.1 [85]. We used the minimum number of observations on each side of the threshold split that is required by TITAN (n = 5). Because our data set was very large, we specified 250 permutations to compute z scores and diagnostic indices as suggested by Baker and King [45].

## Results

### Breeding Bird Survey

**Figure 2:**
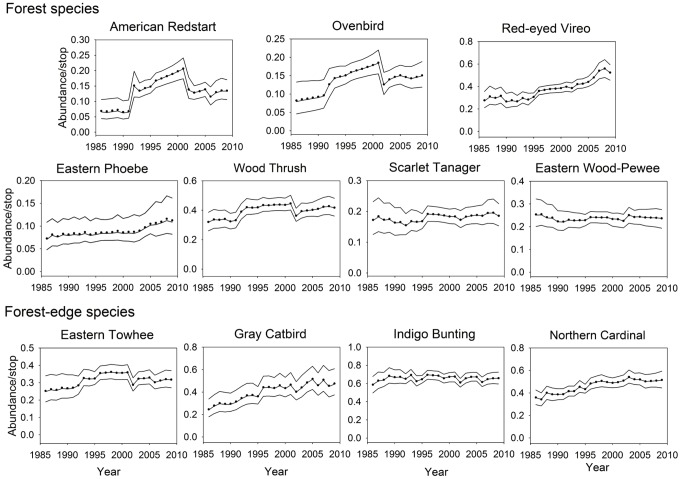
Time series of mean abundance adjusted for missing observations and observer differences. Lines indicate posterior median (line nearly coincident with the circles) with 95% confidence intervals.

There were 2481 detections on the 125 selected survey stops between 1986 and 2009. The most common species was the Indigo Bunting (1108 detections) and the least common was the Eastern Phoebe (190 detections; Table 1). Forest-edge species were the more abundant group (average of 1094 individuals per species) compared to the forest species (525 individual counts per species). Annual mean adjusted abundances (i.e., posterior means) showed population trends of selected species between 1986 and 2009 accounting for differences in route, observer, and detection year (Figure 2). The Gray Catbird, Northern Cardinal, American Redstart, Ovenbird, and Red-eyed Vireo showed significant increases in estimated abundance between 1986 and 2009 (Table 1). American Redstart had the highest percent change per year (3.1%). For the other six species, the estimated abundance did not significantly change through the 24-year period.

**Table 1:** Hierarchical-model estimates based on Breeding Bird Survey stops for forest and forest- edge species.

Species	Number of total detections (% of surveys)	Mean adjusted abundance	Trend coefficient	Percent change/year
**Forest species**				
American Redstart (AMRE)	225 (9.1)	0.132±0.015	**0.042**	**3.10**
Ovenbird (OVEN)	248 (10.0)	0.137±0.016	**0.029**	2.70
Red-eyed Vireo (REVI)	632 (25.5)	0.373±0.027	**0.024**	**2.70**
Eastern Phoebe (EAPH)	190 (7.7)	0.090±0.014	0.005	1.80
Wood Thrush (WOTH)	618 (24.9)	0.396±0.027	0.008	1.10
Scarlet Tanager (SCTA)	364 (14.7)	0.180±0.018	–0.004	0.30
Eastern Wood-Pewee (EAWP)	490 (19.8)	0.237±0.018	–0.001	–0.20
**Forest-edge species**				
Gray Catbird (GRCA)	509 (20.5)	0.401±0.048	**0.025**	**2.80**
Northern Cardinal (NOCA)	808 (32.6)	0.461±0.027	**0.022**	**1.50**
Eastern Towhee (EATO)	526 (21.2)	0.313±0.025	0.007	1.00
Indigo Bunting (INBU)	1108 (44.7)	0.657±0.031	–0.006	0.50

American Ornithologists Union alpha codes for English common names are in parenthesis. Trend coefficient represents the slope on a log scale of abundance over time. Values in bold indicate 95% credible intervals.

### Landscape structure around Breeding Bird Survey stops

Landscape composition and configuration changed through time during the period of study, except for 20% of BBS that were inside protected areas (Table 2). For the 400-m radius buffer, amount of forest decreased from 49.2% in 1986 to 41.2% in 2009; whereas, the amount of exurban development increased from 1.7% in 1986 to 6.0% in 2009. Configuration of forest patches also differed among years. Although the number of forest patches remained nearly constant, area-weighted average patch size decreased by a mean of 2.1 ha in the last time period. This decrease in patch size was accompanied by a decrease in forest edge from 1986 to 2009, a decrease in forest interior, an increase in forest fragments, and a decrease in the proximity index. In general, all metrics changed much more in later time periods than early years reflecting the increasing rate of exurban development in the study region.

**Table 2:** Landscape structure surrounding selected Breeding Bird Survey stops (n  =  125) at 400-m and 1-km radius buffer (mean ± sd) for 1986, 1993, 2000, and 2009.

Variables	1986	1993	2000	2009
**400-m radius buffer**				
Forest (%)	49.2±39.3	48.3±39.3	46.2±39.4	41.2±39.2
Exurban development (%)	1.7±2.5	2.1±2.6	3.1±3.4	6.0±6.8
Forest interior (%)	39.8±32.2	38.1±31.9	35.8±31.8	29.3±32.4
Area- weighted average patch size (ha)	22.2±20.8	21.7±20.7	20.6±20.6	18.5±20.5
Forest fragments (%)	23.4±35.7	23.5±35.6	25.1±37.9	31.9±40.9
Number of forest patches (> 0.45 ha)	1.7±1.1	1.7±1.2	1.6±1.2	1.6±1.4
Forest edge (%)	24.1±14.7	24.3±14.8	24.5±16.4	20.7±16.2
Proximity index	25156.8±071.5	23165.1±749.6	14763.0±2712.3	9884.6±4949.1
**1-km radius buffer**				
Forest (%)	51.0±35.7	50.0±35.6	47.9±35.7	42.7±35.8
Exurban development (%)	1.8±1.6	2.2±1.9	3.2±2.6	6.2±5.6
Forest interior (%)	55.6±28.9	53.1±28.9	49.4±30.2	40.1±32.4
Area- weighted average patch size (ha)	134.4±123.5	131.8±123.1	123.2±121.7	111.6±121.3
Forest fragments (%)	10.2±17.8	11.2±19.6	14.4±24.5	19.9±28.8
Number of forest patches (> 0.45 ha)	5.0±4.2	5.0±4.2	5.3±4.3	5.4±4.4
Forest edge (%)	23.6±11.3	24.5±11.8	24.4±12.6	22.5±12.8
Proximity index	25957.0±205.7	23906.8±060.7	15272.4±1243.6	10533.3±4917.0

Similar patterns were observed for the 1-km radius buffer (Table 2). Those differences that did exist can largely be explained by the area effect of the larger buffer. More forest patches were found in the larger 1-km radius buffer, and these patches were generally larger (e.g., area-weighted average patch size in 2009 of 111.6 ha for the 1-km buffer vs. 18.5 ha for 400-m buffer). The larger buffer also contained fewer forest fragments (19.9 vs. 31.9% in 2009), but underwent a greater loss in forest interior from 1986 to 2009 (6.5% for 1-km buffer vs. 4.4% for 400-m buffer).

### Threshold response of bird species to landscape structure

**Figure 3:**
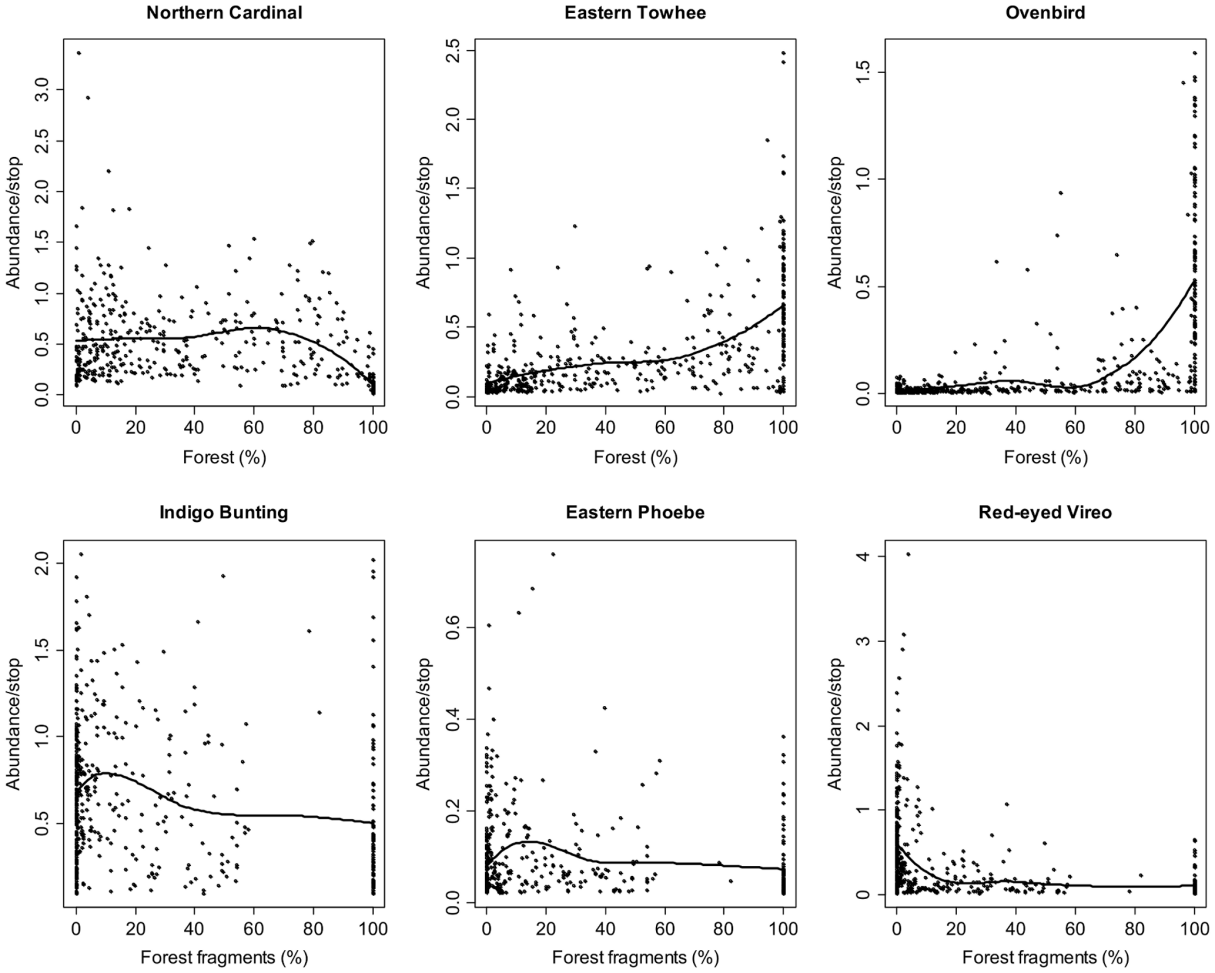
Example relationships between adjusted abundance for species representing the forest and forest-edge groups and selected landscape variables. Landscape composition (e.g., proportion of forest) and landscape configuration (e.g., proportion of forest fragments) in 400-m radius circular areas are depicted. The line represents non-parametric locally weighted polynomial regression curve (loess).

**Figure 4:**
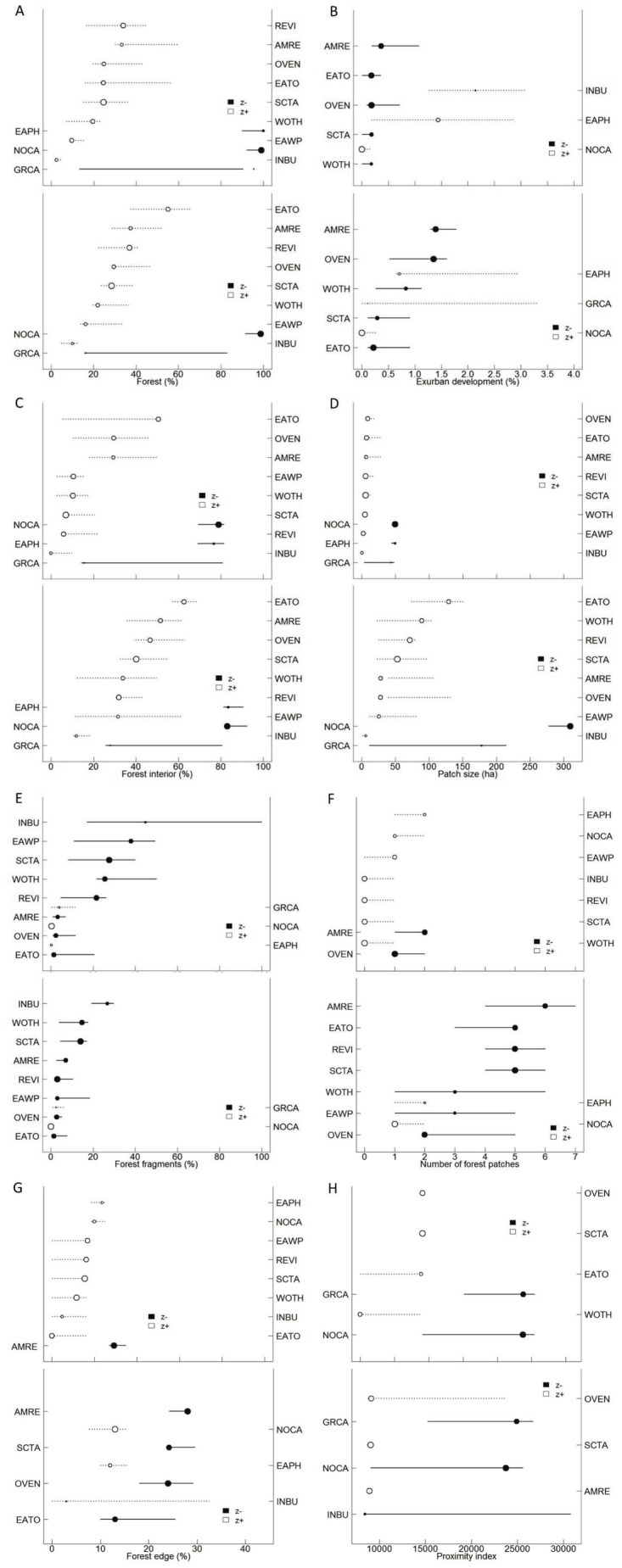
Threshold Indicator Taxa ANalysis (TITAN). Landscape variables were used as predictors of threshold changes in individual bird species in 400-m (top panel) and 1-km radius circular areas (bottom panel) between 1986 and 2009 in north-central Virginia and western Maryland. Only indicator taxa (purity ≥ 0.95 and reliability ≥ 0.95) are plotted in increasing order with respect to their observed change point. Solid circles correspond to negative (z-) indicator taxa (with corresponding species labels on the left axes) and open circles correspond to positive (z+) indicator taxa (with corresponding species labels on the right axes). Circles are sized in proportion to z scores. Lines overlapping each circle represent 5 and 95% percentiles among 250 bootstrap replicates. Landscape variables evaluated were (A) forest, (B) exurban development, (C) forest interior, (D) area-weighted averaged patch size, (E) forest fragments, (F) number of forest patches, (G) forest edge, and (H) proximity index. Taxa IDs correspond to the American Ornithologists Union alpha codes for English common names.

Scatterplots of adjusted counts fitted with a non-parametric locally weighted polynomial regression (loess) model indicated a nonlinear relationship between several of the landscape variables and selected bird species (see examples in Figure 3). In general, forest species exhibited threshold responses to both landscape composition and configuration (Figure 4). For the 400-m radius buffer, most of the forest species were positive indicator taxa for the amount of forest (mean change point: 24.3%), forest interior (15.4%), area-weighted average patch size (5.7 ha), and proximity index (9078). Most of the forest species were negative indicator taxa for the amount of exurban development (0.2%) and proportion of forest fragments (19.7%). American Redstart was the only forest species that responded negatively to forest edge (change point: 29.1%), whereas the rest of the forest species responded positively (mean change point: 16.6%). Eastern Phoebe was the only forest species that declined with amount of forest, proportion of forest interior, and area-weighted average patch size. This species also responded positively to the proportion of forest fragments, though some relationships for this species were of lower reliability (Appendix S1).

Forest-edge species had strong threshold responses to landscape composition and most of the configuration metrics at both extents (Figure 4). For the 400-m radius buffer, for example, all forest-edge species responded positively to the number of forest patches (mean change point: 0.6 patches). Gray Catbird and Northern Cardinal increased sharply with amount of exurban development, proportion of forest fragments, and forest edge (although forest edge was not a reliable indicator for Gray Catbird). These two species responded negatively to the amount of forest, forest interior, area-weighted average patch size, and proximity index (Figure 4). However, Eastern Towhee and Indigo Bunting were positive indicator taxa for the amount of forest, forest interior, area-weighted average patch size, proximity index, and forest edge, and were negative indicator taxa for the proportion of forest fragments. Eastern Towhee was the only forest-edge species that responded negatively to the amount of exurban development and had similar change points to those exhibited by forest species.

Similar patterns in threshold response were observed for the two buffer widths (Figure 4 comparison of top and bottom panels) except for number of forest patches and proportion of forest edge. For these two variables, the direction of the response for roughly half of the species changed with buffer width. For most of the species, the direction of the response was positive for the 400-m radius buffer but negative for the 1-km radius buffer. However, the quality of indicators for the proportion of forest edge was less reliable for the 1-km radius buffer.

The quality of the indicator and confidence around change-point locations varied by extent and by landscape structure variable. For example, the forest species Red-eyed Vireo responded positively to the amount of exurban development. However, the indicator was only moderately reliable for the 400-m radius buffer (reliability  =  0.70; Appendix S1). Reliability also changed with extent of analysis for some species and indicators. For example, the reliability of the response of the forest species Eastern Phoebe to the proximity index was higher within the 400-m radius buffer (reliability  =  0.74) than for the 1-km radius buffer (reliability  =  0.38). Gray Catbird, an edge species, had a positive response to the number of forest patches within the 400-m radius buffer and a negative response within the 1-km radius buffer. However, the reliability for the 1-km radius buffer was poor (reliability  =  0.32). In general, where there were differences in reliability at different extents, the 400-m relationships were more reliable.

Forest species had relatively narrow bootstrapped change-point distributions for most landscape structure characteristics indicating confidence about the existence of a threshold (Figure 4). However, for some landscape structure characteristics, forest species exhibited variable width in the bootstrapped change-point distributions. For example, some species (e.g., Eastern Wood-Pewee) had a sharp response to the amount of forest whereas others (e.g., Red-eyed Vireo) had a more gradual response. In general, forest-edge species (except for Eastern Towhee) had broad bootstrapped change-point distribution suggesting a more gradual response for most landscape structure characteristics.

## Discussion

Our results support the existence of nonlinear responses to habitat loss and fragmentation [37], [41], [43] and variation in sensitivity to alteration of landscape structure due to exurban development depends on species habitat specificity (Figure 4; [41], [86]). For example, species that positively responded to the amount of exurban development (e.g., Northern Cardinal) are often found throughout a range of habitats from shrubby sites in logged and second-growth forests to plantings around buildings [87]. Sensitive species who responded negatively to amount of exurban development (e.g., Wood Thrush) are more frequently found in well-developed deciduous and mixed forests [88].

Despite loss of forest and increase of exurban development, bird sightings significantly increased during the 24-year period for five of the 11 species analyzed. The detection of two of the forest-edge species (Northern Cardinal and Gray Catbird) increased between 1986 and 2009. These species are found in forest edges and clearings, fencerows, abandoned farmland, or residential areas [87], [89]. Thus, more sightings in exurban areas may indicate that these species have been taking advantage of the increased availability of suitable habitats and supplemental feeding provided by landowners [90]. The species also had broad change-point distributions indicating gradual responses to the land-cover change. Although we did not expect to find a threshold response, the direction of the response showed by these species corresponded with their habitat preferences. In other words, these species were indicators of habitat fragmentation due to exurban development (e.g., increased in abundance with increase in forest fragments and decrease in forest interior).

The other three species that experienced abundance increases were forest birds (American Redstart, Red-eyed Vireo, and Ovenbird). This was surprising given documented population declines in other studies for the Red-eyed Vireo and the Ovenbird due to habitat loss and fragmentation (e.g., [32], [56]). American Redstart and Red-eyed Vireo are forest birds but seem to occur more frequently in early and mid-successional forest habitats and even start to decline as forests mature [91]–[93]. Thus, the type of forest disturbance associated with exurban development may benefit these species. The larger temporal and spatial scale regional regrowth of eastern forests due to farmland abandonment since the early twentieth century [94]–[96] also may explain the slight increase in abundance of these species. However, all three of the species showed a strong threshold response to amount of forest, suggesting that they are sensitive to reduced forest cover. It is important to note that the amount of forest of more than 45% of survey stops in 2009 were above the identified thresholds at both extents for American Redstart, Red-eyed Vireo, and Ovenbird. Thus, it seems that abundance increase is occurring disproportionately in relatively intact forests (e.g., protected areas) confounding any negative effects that forest decline [32] in exurban areas may have, though further assessment is required to confirm this assertion.

Although species showed similar response patterns at both extents, for two of the landscape configuration variables (number of patches and forest edge), the direction of the response changed with the extent. Similar results were found by Smith [97] who demonstrate that fragmentation effects depend on the landscape extent considered. Thus, the extent should be explicitly accounted for when evaluating the effects of these two metrics on forest birds. In general, the 400-m relationships were deemed more reliable by the TITAN threshold analysis indicating that more local change processes had a greater effect on species occurrence and relative abundances.

Although the majority of species responses were consistent with our classification regarding habitat preferences, there were two species (Eastern Phoebe and Eastern Towhee) whose response did not correspond to the assigned group. Eastern Phoebe is generally a woodland species [98] and was classified as a forest species. However, this species had threshold responses similar to those exhibited by forest-edge species for most of the landscape structure variables. This may be explained by nest placement preferences. Eastern Phoebe is mostly constrained by availability of suitable nest sites [98] and nests are often located on bridges, culverts, buildings, and rock outcrops in the vicinity of water [99]. Change in landscape structure due to exurban development may benefit this species, but further monitoring of its population is recommended. In contrast, Eastern Towhee exhibited a response similar to those showed by forest species. This species is thought of as an edge-associated generalist and places its nests on or above ground, usually at 1.5 m in shrubby areas [100]. However, these results suggest that Eastern Towhees may be more sensitive to habitat change due to exurban development than previously expected. Alternatively, Eastern Towhees might be more susceptible to increased predation pressure from free-ranging domestic cats common in exurban development [101], [102].

The threshold responses that we detected for selected forest bird species indicate that species were affected in a nonlinear fashion by changes in landscape composition and configuration. However, the thresholds observed may not necessarily be similar for forest bird communities as a whole. In addition, threshold responses detected should not be used as a point below which a population will not persist [103] but rather as guidelines for management practices in areas prone to exurban development.

Given the wide range of threshold values observed in this study (e.g., threshold response to the amount of forest ranged between 9.6 and 33.9% for the 400-m radius buffer), it is problematic to suggest generic recommendations on how to best conserve forest birds in exurban areas. Exurban development is creating habitats that suit forest-edge species, and the main risk is at the other end of the spectrum for the forest species that require large amount of continuous forest cover. Incorporating threshold response in conservation planning might focus on maintaining forested habitats targeted towards the most sensitive species. For example, exurban areas can be managed to retain forest conditions close to the identified thresholds in species occurrence and relative abundance for the most sensitive of selected forest birds such as Red-Eyed Vireo, and in this way other forest birds would also be protected.

It is important to note that the BBS is poor for surveying sensitive forest species with large area requirements. As a result, this analysis considered species that are dependent on forests, but not some of those species that might have been especially sensitive to forest loss (e.g., Kentucky Warbler). Therefore, management efforts targeting the maintenance of larger forest patches as exurban development continues will also benefit some of these other sensitive forest-dependent species. This could be achieved through easements or more focused management for forest birds within existing or new protected areas. The value of high-quality potential source habitat is suggested by the unchanged or even increasing abundance of many of the forest species, although they exhibit negative threshold responses to many of the predictor variables when the entire spatial-temporal dataset is considered. Additional monitoring work, perhaps within the region’s protected areas [104], could expand beyond the BBS roadside surveys to account for some of the limitations of its design.

## Conclusion

Rural private lands are being converted to exurban development at high rates in the Mid-Atlantic region and around the world [28], and this trend is likely to continue into the future [5]. Our results show that exurban development is altering forest habitats. Forest birds exhibited a threshold response to landscape structure alteration at both local and landscape extents. The majority of forest birds’ responses could be predicted by their habitat preferences indicating that management practices in exurban areas might target the maintenance of forested habitats (e.g., through easements or more focused management for birds within existing or new protected areas) lest risk broad-scale changes in bird community composition within these landscapes.

## Supporting Information

Appendix S1 **Threshold Indicator Taxa ANalysis (TITAN) results for forest and forest-edge species for 400-m and 1-km radius buffer.**
(DOCX)
