# Supplementary material for: Threshold Responses of Forest Birds to Landscape Changes around Exurban Development
Source: PLoS One. 2013 Jun 24;8(6):e67593. doi: 10.1371/journal.pone.0067593 (PMC3691136; doi:10.1371/journal.pone.0067593)
Supplement: Appendix S1 — Threshold Indicator Taxa ANalysis (TITAN) results for forest and forest-edge species for 400-m and 1-km radius buffer. (DOCX) [file pone.0067593.s001.docx]

Appendix S1. Threshold Indicator Taxa ANalysis (TITAN) results for forest and forest-edge species for 400-m and 1-km radius buffer.

|  | 400m-radius buffer | | | | | | | 1km-radius buffer | | | | | |
| --- | --- | --- | --- | --- | --- | --- | --- | --- | --- | --- | --- | --- | --- |
|  |  |  | Change point | | |  |  |  |  | Change point | | |  |
|  | Indicator | z | Obs. | 5% | 95% | Reliability |  | Indicator | z | Obs. | 5% | 95% | Reliability |
| **Forest (%)** | |  |  |  |  |  |  |  |  |  |  |  |  |
| Forest species | | |  |  |  |  |  |  |  |  |  |  |  |
| AMRE | z+ | 14.91 | 33.15 | 28.23 | 69.35 | 1.00 |  | z+ | 15.98 | 37.45 | 24.79 | 57.14 | 1.00 |
| OVEN | z+ | 18.46 | 24.69 | 16.97 | 49.69 | 1.00 |  | z+ | 18.18 | 29.41 | 25.12 | 54.55 | 1.00 |
| REVI | z+ | 22.08 | 33.93 | 15.16 | 49.69 | 1.00 |  | z+ | 22.85 | 36.79 | 19.27 | 43.45 | 1.00 |
| EAPH | z- | 10.52 | 100.00 | 11.14 | 100.00 | 0.94 |  | z- | 9.75 | 99.95 | 12.59 | 100.00 | 0.71 |
| WOTH | z+ | 19.93 | 19.61 | 4.57 | 28.17 | 1.00 |  | z+ | 18.20 | 21.97 | 16.67 | 37.91 | 1.00 |
| SCTA | z+ | 26.69 | 24.64 | 14.75 | 39.52 | 1.00 |  | z+ | 26.40 | 28.36 | 22.85 | 41.30 | 1.00 |
| EAWP | z+ | 19.50 | 9.64 | 8.11 | 17.67 | 1.00 |  | z+ | 15.26 | 16.22 | 12.10 | 38.36 | 1.00 |
| Forest-edge species | | |  |  |  |  |  |  |  |  |  |  |  |
| GRCA | z- | 5.65 | 95.57 | 12.32 | 94.27 | 0.99 |  | z- | 5.42 | 16.22 | 14.98 | 84.17 | 1.00 |
| NOCA | z- | 26.62 | 98.93 | 89.88 | 100.00 | 1.00 |  | z- | 25.56 | 98.68 | 88.77 | 100.00 | 1.00 |
| EATO | z+ | 19.68 | 24.64 | 14.97 | 68.77 | 1.00 |  | z+ | 18.80 | 55.04 | 29.21 | 69.74 | 1.00 |
| INBU | z+ | 14.72 | 2.32 | 1.16 | 6.08 | 1.00 |  | z+ | 11.70 | 9.98 | 2.99 | 12.94 | 1.00 |
| **Exurban development (%)** | | | |  |  |  |  |  |  |  |  |  |  |
| Forest species | | |  |  |  |  |  |  |  |  |  |  |  |
| AMRE | z- | 12.35 | 0.36 | 0.18 | 1.43 | 1.00 |  | z- | 12.97 | 1.40 | 1.21 | 1.89 | 1.00 |
| OVEN | z- | 14.21 | 0.18 | 0.00 | 0.85 | 1.00 |  | z- | 13.85 | 1.35 | 0.28 | 1.71 | 1.00 |
| REVI | z+ | 5.46 | 11.94 | 0.00 | 12.31 | 0.70 |  | z+ | 6.59 | 4.50 | 0.19 | 7.97 | 0.87 |
| EAPH | z+ | 11.72 | 1.44 | 0.18 | 3.28 | 1.00 |  | z+ | 12.52 | 0.71 | 0.57 | 3.08 | 1.00 |
| WOTH | z- | 7.62 | 0.18 | 0.00 | 4.11 | 0.94 |  | z- | 8.70 | 0.83 | 0.22 | 1.28 | 1.00 |
| SCTA | z- | 9.68 | 0.18 | 0.00 | 0.18 | 0.99 |  | z- | 9.83 | 0.29 | 0.00 | 1.03 | 0.98 |
| EAWP | z- | 5.86 | 0.00 | 0.00 | 8.63 | 0.86 |  | z- | 5.82 | 0.31 | 0.00 | 4.40 | 0.92 |
| Forest-edge species | | |  |  |  |  |  |  |  |  |  |  |  |
| GRCA | z+ | 3.02 | 0.00 | 0.00 | 9.01 | 0.84 |  | z+ | 4.01 | 0.11 | 0.00 | 3.76 | 0.98 |
| NOCA | z+ | 17.07 | 0.00 | 0.00 | 0.18 | 1.00 |  | z+ | 27.10 | 0.00 | 0.00 | 0.29 | 1.00 |
| EATO | z- | 13.50 | 0.18 | 0.00 | 0.36 | 1.00 |  | z- | 14.14 | 0.22 | 0.02 | 1.17 | 1.00 |
| INBU | z+ | 4.61 | 2.14 | 0.71 | 9.09 | 0.94 |  | z+ | 2.73 | 4.41 | 0.11 | 7.81 | 0.62 |
| **Forest interior (%)** | | |  |  |  |  |  |  |  |  |  |  |  |
| Forest species | | |  |  |  |  |  |  |  |  |  |  |  |
| AMRE | z+ | 14.90 | 29.35 | 17.28 | 51.58 | 1.00 |  | z+ | 15.44 | 51.57 | 33.32 | 62.69 | 1.00 |
| OVEN | z+ | 16.61 | 29.51 | 8.31 | 50.86 | 1.00 |  | z+ | 16.34 | 46.55 | 38.31 | 66.01 | 1.00 |
| REVI | z+ | 17.78 | 5.88 | 3.29 | 28.07 | 1.00 |  | z+ | 17.58 | 32.01 | 29.28 | 47.99 | 1.00 |
| EAPH | z- | 9.73 | 76.69 | 66.39 | 81.55 | 0.99 |  | z- | 10.13 | 83.51 | 80.60 | 92.37 | 1.00 |
| WOTH | z+ | 20.72 | 10.20 | 1.48 | 20.36 | 1.00 |  | z+ | 14.84 | 33.81 | 11.19 | 62.51 | 1.00 |
| SCTA | z+ | 23.17 | 6.85 | 4.96 | 25.12 | 1.00 |  | z+ | 21.29 | 40.08 | 31.06 | 57.38 | 1.00 |
| EAWP | z+ | 19.64 | 10.53 | 1.98 | 16.30 | 1.00 |  | z+ | 12.53 | 31.48 | 10.86 | 63.11 | 1.00 |
| Forest-edge species | | |  |  |  |  |  |  |  |  |  |  |  |
| GRCA | z- | 5.95 | 15.67 | 12.28 | 81.49 | 1.00 |  | z- | 5.07 | 27.88 | 23.42 | 81.74 | 1.00 |
| NOCA | z- | 27.08 | 78.87 | 67.28 | 81.61 | 1.00 |  | z- | 26.64 | 83.02 | 79.14 | 92.44 | 1.00 |
| EATO | z+ | 18.10 | 50.49 | 2.00 | 56.02 | 1.00 |  | z+ | 16.97 | 62.58 | 54.54 | 70.31 | 1.00 |
| INBU | z+ | 11.68 | 0.00 | 0.00 | 10.50 | 1.00 |  | z+ | 10.39 | 11.91 | 7.12 | 23.46 | 1.00 |
| **Area-weighted average patch size (ha)** | | | | |  |  |  |  |  |  |  |  |  |
| Forest species | | |  |  |  |  |  |  |  |  |  |  |  |
| AMRE | z+ | 15.24 | 6.55 | 7.26 | 29.42 | 1.00 |  | z+ | 15.43 | 28.30 | 36.48 | 125.34 | 1.00 |
| OVEN | z+ | 18.16 | 9.13 | 4.77 | 26.44 | 1.00 |  | z+ | 16.87 | 27.85 | 33.63 | 150.75 | 1.00 |
| REVI | z+ | 21.27 | 5.69 | 3.46 | 20.04 | 1.00 |  | z+ | 21.24 | 71.36 | 21.01 | 98.24 | 1.00 |
| EAPH | z- | 9.70 | 49.28 | 19.57 | 50.11 | 0.95 |  | z- | 9.89 | 295.16 | 11.54 | 313.66 | 0.87 |
| WOTH | z+ | 22.31 | 4.89 | 2.02 | 7.49 | 1.00 |  | z+ | 17.28 | 89.31 | 19.02 | 113.37 | 1.00 |
| SCTA | z+ | 24.75 | 5.69 | 3.59 | 13.37 | 1.00 |  | z+ | 23.51 | 52.89 | 17.56 | 109.40 | 1.00 |
| EAWP | z+ | 17.78 | 2.40 | 1.94 | 7.46 | 1.00 |  | z+ | 14.40 | 25.49 | 9.33 | 99.18 | 1.00 |
| Forest-edge species | | |  |  |  |  |  |  |  |  |  |  |  |
| GRCA | z- | 5.77 | 43.79 | 3.01 | 48.96 | 0.99 |  | z- | 6.20 | 177.78 | 8.62 | 234.90 | 1.00 |
| NOCA | z- | 26.81 | 49.55 | 44.66 | 49.86 | 1.00 |  | z- | 25.91 | 309.60 | 264.68 | 312.12 | 1.00 |
| EATO | z+ | 19.47 | 7.12 | 2.74 | 31.99 | 1.00 |  | z+ | 17.47 | 129.26 | 69.60 | 165.96 | 1.00 |
| INBU | z+ | 12.54 | 0.33 | 0.19 | 1.69 | 1.00 |  | z+ | 9.31 | 6.18 | 1.17 | 7.95 | 1.00 |
| **Forest fragments (%)** | | |  |  |  |  |  |  |  |  |  |  |  |
| Forest species | | |  |  |  |  |  |  |  |  |  |  |  |
| AMRE | z- | 14.77 | 3.22 | 0.60 | 7.67 | 1.00 |  | z- | 15.74 | 7.09 | 1.79 | 8.64 | 1.00 |
| OVEN | z- | 16.26 | 2.33 | 0.65 | 15.53 | 1.00 |  | z- | 17.11 | 2.67 | 1.16 | 6.72 | 1.00 |
| REVI | z- | 19.09 | 21.51 | 3.79 | 35.60 | 1.00 |  | z- | 21.57 | 2.98 | 2.71 | 10.98 | 1.00 |
| EAPH | z+ | 6.97 | 0.20 | 0.00 | 0.61 | 0.98 |  | z+ | 9.78 | 0.02 | 0.00 | 19.40 | 0.83 |
| WOTH | z- | 18.53 | 25.68 | 15.96 | 53.97 | 1.00 |  | z- | 17.97 | 14.76 | 2.94 | 18.21 | 1.00 |
| SCTA | z- | 21.71 | 27.63 | 4.99 | 42.86 | 1.00 |  | z- | 21.97 | 14.01 | 3.36 | 18.08 | 1.00 |
| EAWP | z- | 16.01 | 38.00 | 4.22 | 55.40 | 1.00 |  | z- | 14.31 | 2.98 | 2.03 | 19.36 | 1.00 |
| Forest-edge species | | |  |  |  |  |  |  |  |  |  |  |  |
| GRCA | z+ | 6.65 | 3.97 | 0.00 | 26.11 | 1.00 |  | z+ | 5.45 | 2.52 | 0.16 | 19.40 | 1.00 |
| NOCA | z+ | 17.03 | 0.21 | 0.00 | 0.58 | 1.00 |  | z+ | 25.06 | 0.02 | 0.00 | 0.12 | 1.00 |
| EATO | z- | 16.44 | 1.30 | 0.59 | 39.88 | 1.00 |  | z- | 16.78 | 1.32 | 0.91 | 9.79 | 1.00 |
| INBU | z- | 7.98 | 44.77 | 15.40 | 100.00 | 0.99 |  | z- | 12.97 | 26.75 | 17.50 | 31.77 | 1.00 |
| **Number of forest patches > 0.45 ha** | | | |  |  |  |  |  |  |  |  |  |  |
| Forest species | | |  |  |  |  |  |  |  |  |  |  |  |
| AMRE | z- | 12.47 | 2.00 | 1.00 | 2.00 | 0.99 |  | z- | 14.48 | 6.00 | 3.45 | 7.00 | 1.00 |
| OVEN | z- | 14.32 | 1.00 | 1.00 | 2.00 | 1.00 |  | z- | 16.29 | 2.00 | 2.00 | 6.00 | 1.00 |
| REVI | z+ | 14.27 | 0.00 | 0.00 | 1.00 | 1.00 |  | z- | 16.05 | 5.00 | 4.00 | 6.28 | 1.00 |
| EAPH | z+ | 8.79 | 2.00 | 1.00 | 2.00 | 1.00 |  | z+ | 6.26 | 2.00 | 1.00 | 3.00 | 1.00 |
| WOTH | z+ | 16.24 | 0.00 | 0.00 | 1.00 | 1.00 |  | z- | 9.56 | 3.00 | 1.00 | 6.00 | 0.99 |
| SCTA | z+ | 14.62 | 0.00 | 0.00 | 1.00 | 1.00 |  | z- | 17.05 | 5.00 | 3.00 | 6.00 | 1.00 |
| EAWP | z+ | 11.96 | 1.00 | 0.00 | 1.00 | 0.98 |  | z- | 9.00 | 3.00 | 1.00 | 6.00 | 1.00 |
| Forest-edge species | | |  |  |  |  |  |  |  |  |  |  |  |
| GRCA | z+ | 4.27 | 1.00 | 1.00 | 4.00 | 0.78 |  | z- | 5.44 | 13.00 | 1.00 | 13.00 | 0.32 |
| NOCA | z+ | 9.11 | 1.00 | 1.00 | 2.00 | 1.00 |  | z+ | 17.72 | 1.00 | 1.00 | 2.00 | 1.00 |
| EATO | z+ | 11.23 | 0.50 | 0.00 | 2.00 | 0.85 |  | z- | 14.78 | 5.00 | 2.00 | 5.00 | 1.00 |
| INBU | z+ | 13.93 | 0.00 | 0.00 | 1.00 | 1.00 |  | z+ | 3.28 | 0.00 | 0.00 | 14.00 | 0.92 |
| **Forest edge (%)** | | |  |  |  |  |  |  |  |  |  |  |  |
| Forest species | | |  |  |  |  |  |  |  |  |  |  |  |
| AMRE | z- | 12.16 | 29.12 | 24.18 | 35.78 | 0.98 |  | z- | 14.97 | 28.02 | 23.19 | 29.16 | 1.00 |
| OVEN | z+ | 13.72 | 16.08 | 0.00 | 25.58 | 0.84 |  | z- | 15.51 | 23.94 | 16.01 | 30.38 | 1.00 |
| REVI | z+ | 16.36 | 16.08 | 0.00 | 16.87 | 1.00 |  | z- | 9.43 | 28.27 | 6.81 | 32.85 | 0.87 |
| EAPH | z+ | 9.75 | 23.53 | 18.31 | 25.72 | 1.00 |  | z+ | 11.49 | 12.03 | 9.68 | 16.06 | 1.00 |
| WOTH | z+ | 18.41 | 11.60 | 0.00 | 16.75 | 1.00 |  | z+ | 8.80 | 5.96 | 0.00 | 28.55 | 0.46 |
| SCTA | z+ | 19.79 | 15.42 | 0.00 | 16.76 | 1.00 |  | z- | 13.53 | 24.16 | 22.91 | 30.61 | 0.97 |
| EAWP | z+ | 16.84 | 16.73 | 0.00 | 16.88 | 1.00 |  | z- | 6.83 | 27.73 | 0.00 | 36.53 | 0.82 |
| Forest-edge species | | |  |  |  |  |  |  |  |  |  |  |  |
| GRCA§ | z- | 1.80 | 0.00 | 0.00 | 48.89 | 0.66 |  | z+ | 3.04 | 29.15 | 6.81 | 41.47 | 0.66 |
| NOCA | z+ | 11.41 | 19.83 | 18.35 | 25.94 | 0.98 |  | z+ | 18.68 | 13.02 | 7.56 | 16.01 | 1.00 |
| EATO | z+ | 16.11 | 0.00 | 0.00 | 16.70 | 1.00 |  | z- | 14.06 | 13.02 | 9.42 | 26.19 | 1.00 |
| INBU | z+ | 10.33 | 4.73 | 0.00 | 17.46 | 0.98 |  | z+ | 5.33 | 2.93 | 0.00 | 34.48 | 0.98 |
| **Proximity index** | | | |  |  |  |  |  |  |  |  |  |  |
| Forest species | | |  |  |  |  |  |  |  |  |  |  |  |
| AMRE | z+ | 8.45 | 9075.3 | 9008.0 | 23749.3 | 0.92 |  | z+ | 8.08 | 8910.6 | 8688.6 | 9108.2 | 0.97 |
| OVEN | z+ | 9.03 | 9075.3 | 8993.5 | 9108.2 | 1.00 |  | z+ | 7.65 | 9108.2 | 8711.5 | 23749.3 | 0.96 |
| REVI | z+ | 7.71 | 9086.1 | 8829.7 | 12855.1 | 0.94 |  | z+ | 6.14 | 9023.7 | 8737.5 | 19193.7 | 0.46 |
| EAPH§ | z+ | 3.65 | 25818.9 | 0.0 | 26172.4 | 0.74 |  | z+ | 3.24 | 26042.0 | 8420.1 | 26712.9 | 0.38 |
| WOTH | z+ | 7.45 | 0.0 | 0.0 | 9076.1 | 0.97 |  | z+ | 2.74 | 23739.6 | 8409.9 | 36803.3 | 0.53 |
| SCTA | z+ | 10.46 | 9075.3 | 8993.5 | 9108.2 | 1.00 |  | z+ | 8.65 | 9023.7 | 8610.9 | 9108.2 | 1.00 |
| EAWP§ | z+ | 5.61 | 4376.0 | 0.0 | 26495.8 | 0.66 |  | z- | 3.49 | 28483.6 | 8429.7 | 28704.0 | 0.77 |
| Forest-edge species | | |  |  |  |  |  |  |  |  |  |  |  |
| GRCA | z- | 5.72 | 23749.3 | 9108.2 | 25507.3 | 0.98 |  | z- | 6.01 | 24914.9 | 9108.2 | 27801.1 | 1.00 |
| NOCA | z- | 6.36 | 23701.4 | 9048.2 | 25674.9 | 0.99 |  | z- | 7.12 | 23747.8 | 8820.7 | 25780.0 | 1.00 |
| EATO | z+ | 6.66 | 8865.9 | 0.0 | 9108.2 | 0.97 |  | z+ | 5.38 | 8688.6 | 8637.5 | 27322.3 | 0.84 |
| INBU | z+ | 7.21 | 4376.1 | 0.0 | 25811.3 | 0.78 |  | z- | 3.10 | 8449.8 | 8394.4 | 32095.0 | 0.98 |

*Note:* TITAN observed change points (obs.) and bootstrap confidence intervals (among 250 simulation iterations) correspond to the value of independent variables resulting in the largest z scores for each taxon. Reliability is the mean proportion of p-values ≤ 0.05 among 250 simulation iterations.

Taxa IDs correspond to the American Ornithologist´s Union alpha codes for English common names.

§Non-significant species at a 0.05 level.
